# Toxic Shock Syndrome: Rare but Deadly

**DOI:** 10.7759/cureus.69220

**Published:** 2024-09-11

**Authors:** Jorge Mendes, Miguel G Santos, Simone Costa, Luísa Pinto, Fernando Henriques

**Affiliations:** 1 Intensive Care Unit, Centro Hospitalar de Leiria, Leiria, PRT

**Keywords:** emergency medicine, group a streptococcus, hyperbaric oxygen, necrotizing fasciitis, streptococcus pyogenes, toxic shock

## Abstract

Invasive group A streptococcal (GAS) disease, although rare, has a high mortality and morbidity rate, making early recognition and treatment crucial. Toxic shock syndrome (TSS) and necrotizing fasciitis are the most feared complications and require comprehensive, multidisciplinary treatment. In addition to appropriate support and resuscitation, patient management should include empirical broad-spectrum antibiotic therapy covering gram-negative bacteria, methicillin-resistant *Staphylococcus aureus* (MRSA), and anti-toxin therapy. Early surgical debridement is essential for improving the patient's prognosis, and other treatments, such as immunoglobulin and hyperbaric oxygen therapy (HBOT), also appear to be important.

The authors describe the clinical case of a 31-year-old man with no medical history or risk factors, who developed invasive disease from *Streptococcus pyogenes* with rapid progression to necrotizing fasciitis, TSS, and severe multi-organ dysfunction. His management required intensive care, multiple surgical debridements, admission to the intensive care unit, and targeted as well as supportive therapy. The patient survived, but nearly a year later, he has yet to fully return to a normal life.

## Introduction

*Streptococcus pyogenes*, a group A streptococcus, is an aerobic gram-positive coccus commonly associated with pharyngitis and skin or soft tissue infections. However, it can also cause a wide range of other infections [1]. While mild infections are relatively common, invasive group A streptococcal (GAS) infections are rare and are associated with high morbidity and mortality, even in previously healthy individuals [2]. This infection can occur in patients of any age, but its incidence is higher in older adults and very young children [3]. Invasive infections include lower respiratory tract infections, bacteremia, pregnancy-associated infections, or necrotizing soft tissue infections, with necrotizing fasciitis and progression to toxic shock syndrome (TSS) being the most severe and feared complications, both of which are life-threatening conditions [2,4].

## Case presentation

A previously healthy 31-year-old man presented to the emergency department with a history of less than 48 hours of fever and pain, swelling, and erythema in the mid-third of the right leg. The patient denied any trauma, insect bites, recreational drug use, recent surgery, corticosteroid or nonsteroidal anti-inflammatory drug (NSAID) use, or any other complaints.

Initially, he was assessed by the Internal Medicine team, which described a Glasgow Coma Scale (GCS) score of 15 and tachypnea with a respiratory rate of 24 but without other signs of respiratory distress. He had warm and well-perfused extremities, a blood pressure of 102/58 mmHg, and a heart rate of 115 beats per minute. Blood samples, including blood cultures, were collected, and arterial blood gas analysis showed a pH of 7.36, pO2 98 mmHg, pCO2 28 mmHg, HCO3- 18.2 mmol/L, and lactate 2.9 mmol/L. Fluid therapy was initiated, and 2 grams of ceftriaxone were administered. Because of the rapid deterioration, the intensive care unit (ICU) team was called.

**Figure 1:**
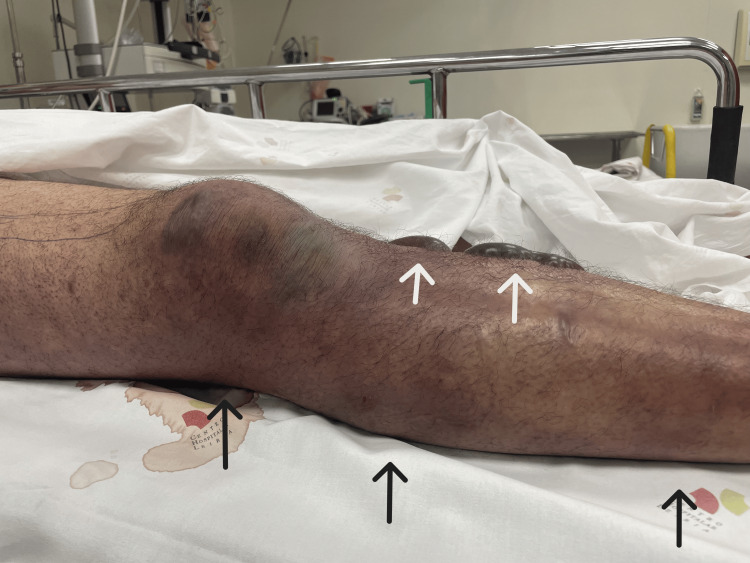
Patient's right leg. The image shows the patient's right leg, displaying hemorrhagic bullae indicated by white arrows, along with marked signs of hypoperfusion and inflammation, including discoloration and edema, in the context of necrotizing fasciitis, highlighted by black arrows.

**Figure 2:**
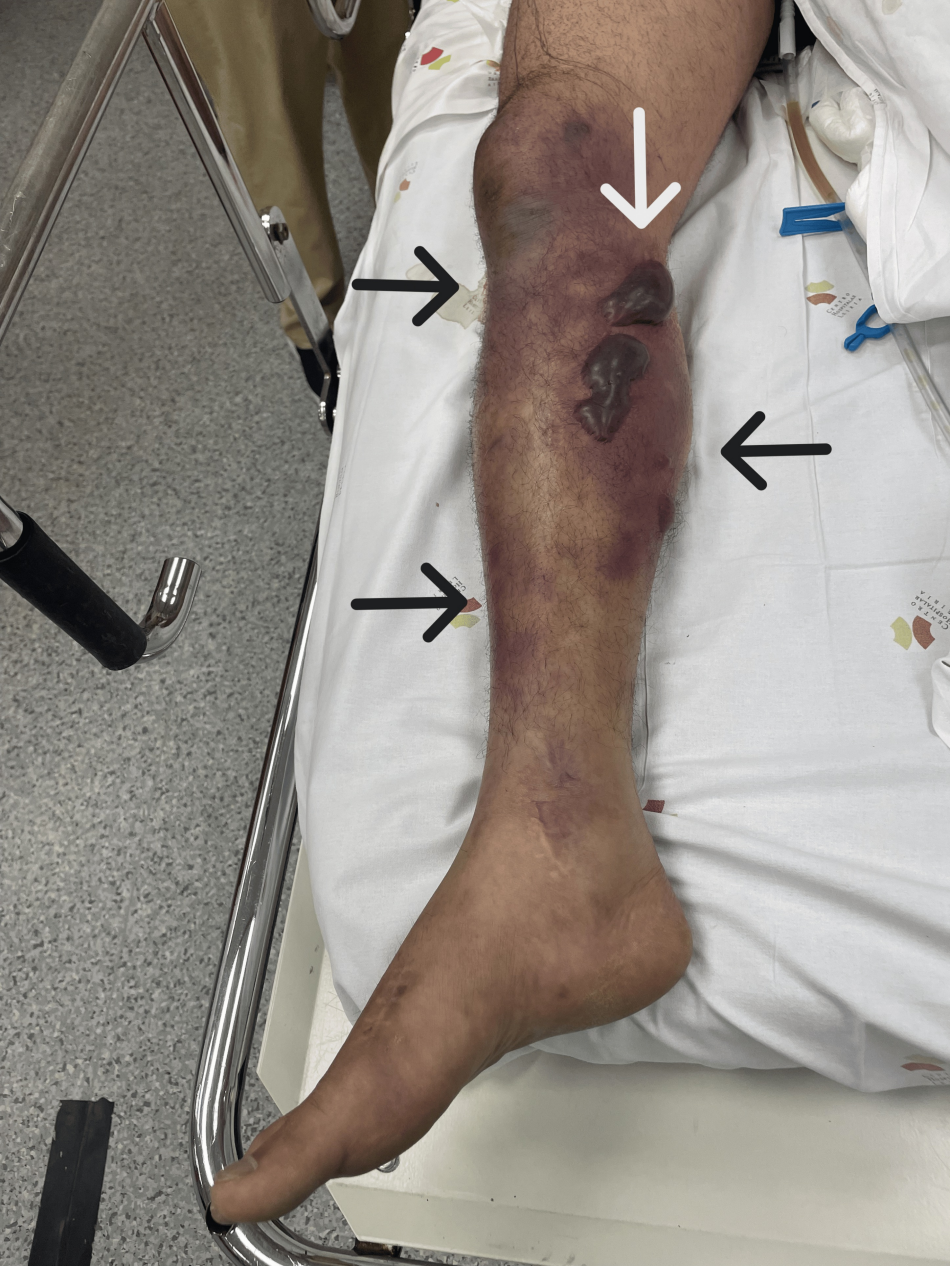
Patient's right leg. The image shows the patient's right leg, displaying hemorrhagic bullae indicated by a white arrow, along with marked signs of hypoperfusion and inflammation, including discoloration and edema, in the context of necrotizing fasciitis, highlighted by black arrows.

Upon our evaluation, the patient's condition had worsened, presenting a GCS of 11 (eye 2, verbal 4, motor 5). He was tachypneic with a respiratory rate of 40 breaths per minute and diaphoretic, with poorly perfused extremities, mottling grade 3, a blood pressure of 82/38 mmHg after receiving 2.5 liters of crystalloids, a heart rate of 140 beats per minute, and a fever of 38.5°C despite having received acetaminophen. The right leg showed significant worsening compared to the initial description by the Internal Medicine team, with hemorrhagic bullae (Figure 1 and Figure 2). Repeat arterial blood gas analysis on 28% FiO2 showed a pH of 7.24, pO2 81 mmHg, pCO2 21 mmHg, HCO3- 13.9 mmol/L, and lactate 6.8 mmol/L.

**Figure 3:**
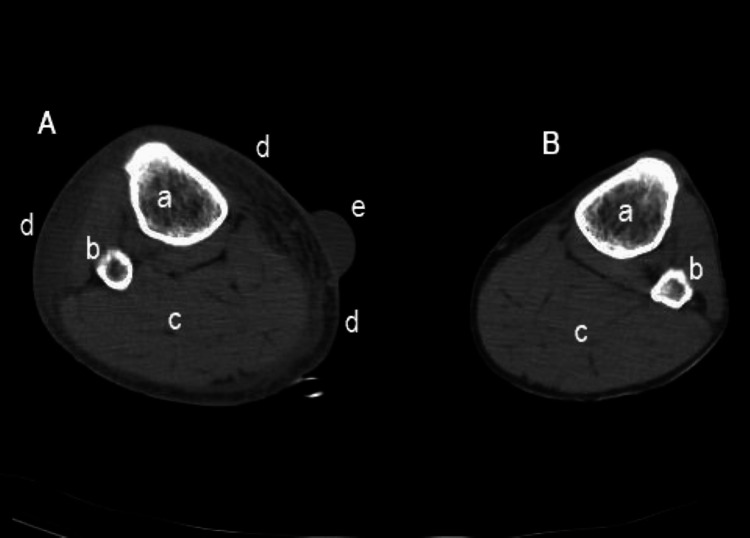
CT scan of the lower limbs. The image shows a CT scan of the lower limbs below the knee, revealing tissue infiltration with edema and a hemorrhagic bulla in the right leg. A: right leg; B: left leg; a: tibia; b: fibula; c: muscle tissue; d: extensive edema infiltrating the subcutaneous tissues; e: hemorrhagic bulla

**Figure 4:**
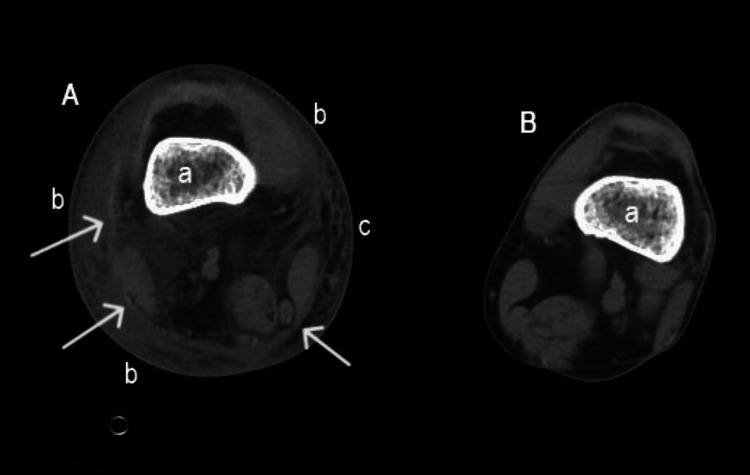
CT scan of the lower limbs. The image shows a CT scan of the lower limbs just above the knee, revealing marked tissue infiltration with edema, fascial thickening, and mild subcutaneous emphysema in the right leg. A: right leg; B: left leg; a: femur; b: extensive subcutaneous tissue edema extending circumferentially around the entire leg; c: mild subcutaneous emphysema; white arrows: fascial thickening

The initial complete blood count revealed leukocytosis with neutrophilia and thrombocytopenia. Blood chemistries showed acute kidney injury, elevated liver enzymes, rhabdomyolysis, and elevated C-reactive protein. The most significant blood test results are presented below in Table 1. A CT scan of the lower limbs showed marked tissue edema of the right leg and hemorrhagic bullae (Figure 3 and Figure 4).

**Table 1:** Patient's blood test results. AST: aspartate transferase; ALT: alanine transaminase; ALP: alkaline phosphatase; GGT: gamma-glutamyl transferase; LDH: lactate dehydrogenase; CK: creatine kinase

Blood analysis	Result
Leukocytes	19.8 g/dL
Neutrophils	15.1 g/dL
Hemoglobin	12.8 g/dL
Platelets	92 g/dL
Creatinine	2.77 mg/dL
AST	292 U/L
ALT	232 U/L
ALP	378 U/L
GGT	341 U/L
Total bilirubin	2.1 mg/dL
Direct bilirubin	0.8 mg/dL
LDH	958 U/L
CK	3284 U/L
Myoglobin	5422 U/L
C-reactive protein	438.6 mg/L

The patient underwent orotracheal intubation, and central venous access, arterial line, nasogastric tube, and urinary catheter were placed. Norepinephrine infusion was started, along with hydrocortisone, albumin, crystalloids, 9 grams of piperacillin/tazobactam, 900 mg of clindamycin, and 600 mg of linezolid. The case was discussed with General Surgery, which evaluated the patient and, given the probable diagnosis of necrotizing fasciitis or myositis, decided to take him to the operating room for debridement. For just over an hour, the patient remained in the emergency room awaiting transfer to the operating room, during which time there were progression of the inflammatory signs above the knee, doubling of the hemorrhagic bullae in size, development of subcutaneous emphysema, and the appearance of a maculopapular rash on the trunk.

**Figure 5:**
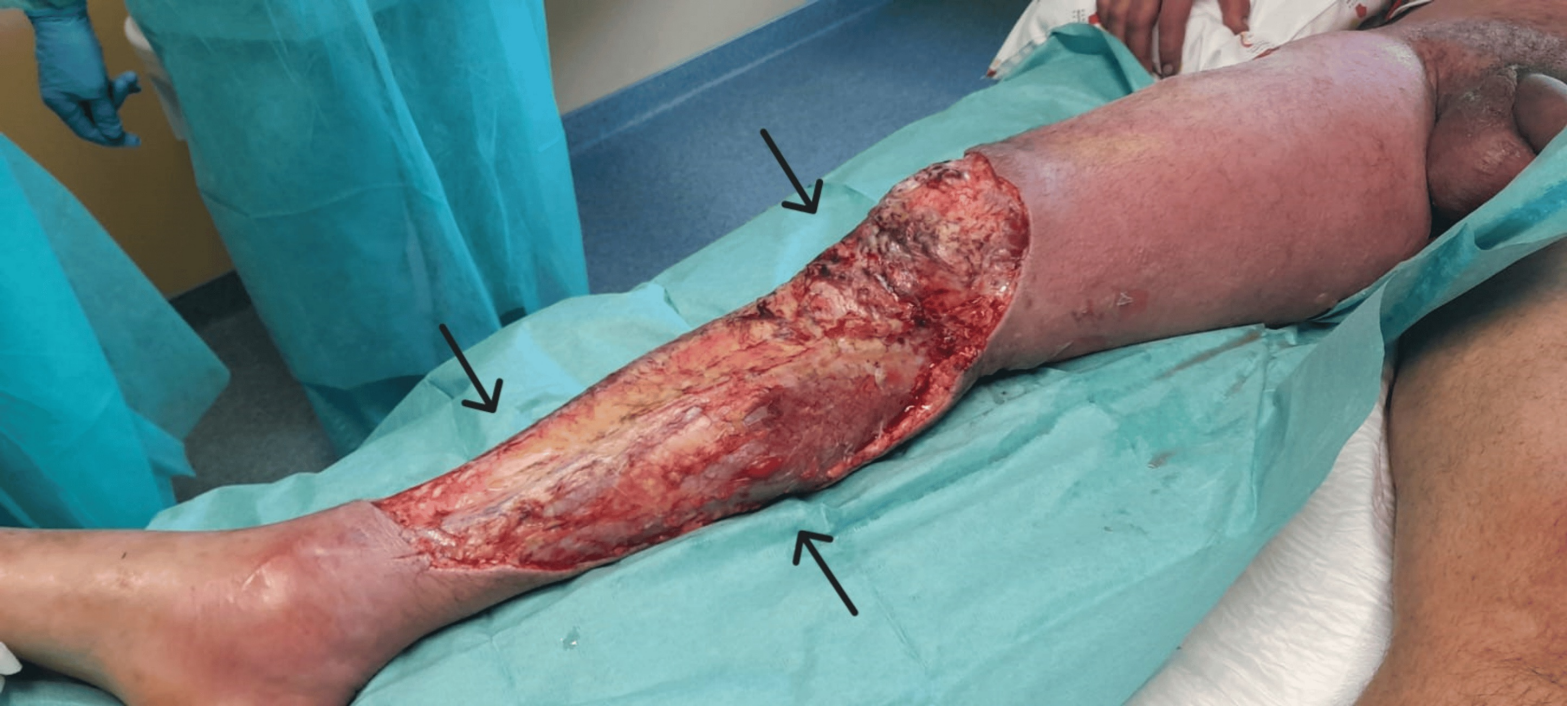
Right leg after the first surgical debridement (black arrows).

After debridement in the operating room (Figure 5), the patient was admitted to the ICU, where renal replacement therapy was initiated due to anuria since admission and a pH of 7.04 with bicarbonate of 8.1, with bicarbonate supplementation in the therapy as well as intravenous bicarbonate administration. Additionally, negative pressure wound therapy was implemented, and intravenous immunoglobulin therapy was also initiated at 1 gram per kilogram on the first day, followed by 0.5 grams per kilogram on the next two days. Despite these measures, the dose of norepinephrine more than doubled, reaching approximately 2.6 μg/kg/min at the time of admission, leading to the initiation of vasopressin and continued resuscitation with crystalloids. The patient remained on piperacillin/tazobactam 18 grams, clindamycin 2.7 grams, and linezolid 1.2 grams per day, along with hydrocortisone, albumin, and other supportive therapies. The medications were adjusted for renal function after 48 hours. On the third day of hospitalization, linezolid was discontinued after *Streptococcus pyogenes* was isolated from blood cultures, which was later also isolated from the surgically removed tissue from the patient's leg. Serological tests for hepatitis B, hepatitis C, and human immunodeficiency virus (HIV) were negative.

**Figure 6:**
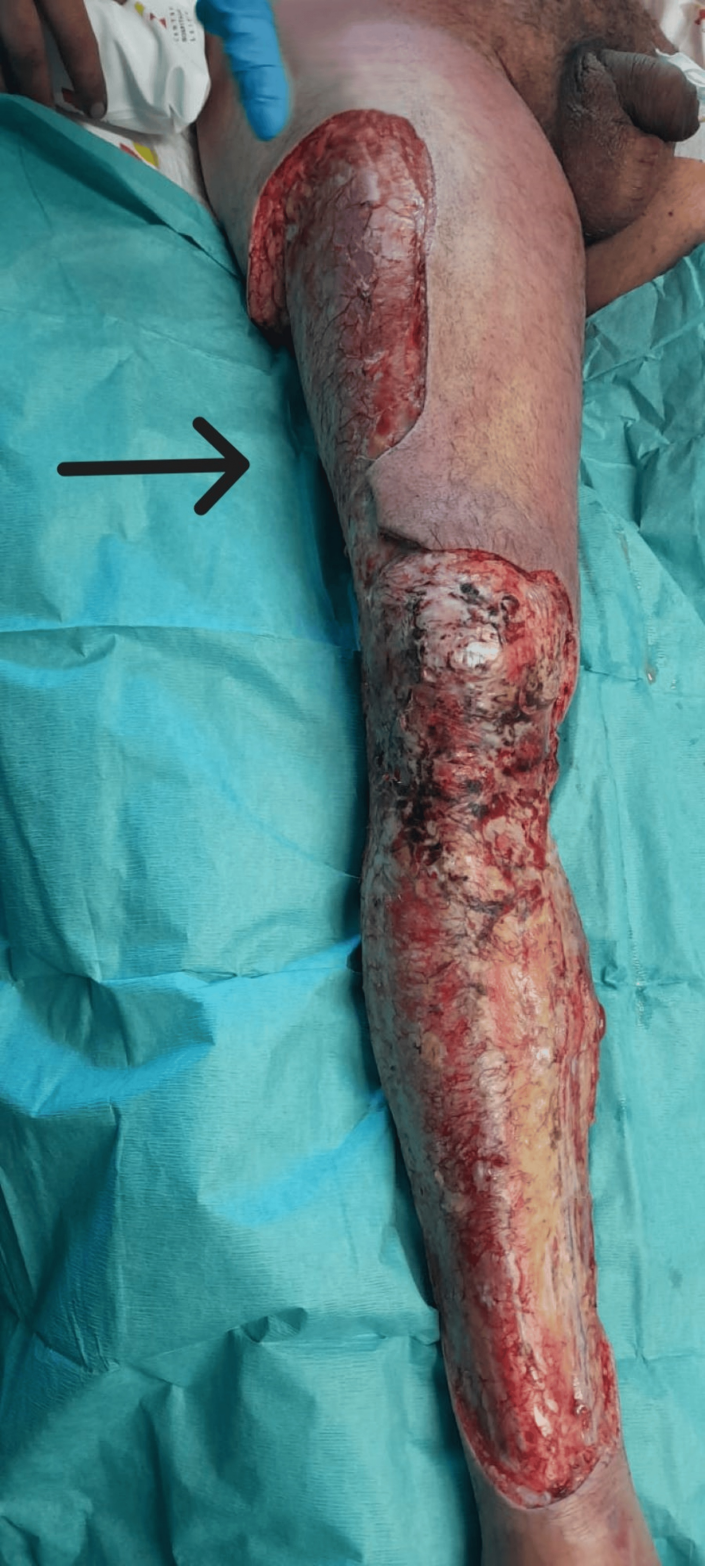
Progression in inflammatory signs to the lateral region of the right thigh requiring surgical debridement (black arrow).

**Figure 7:**
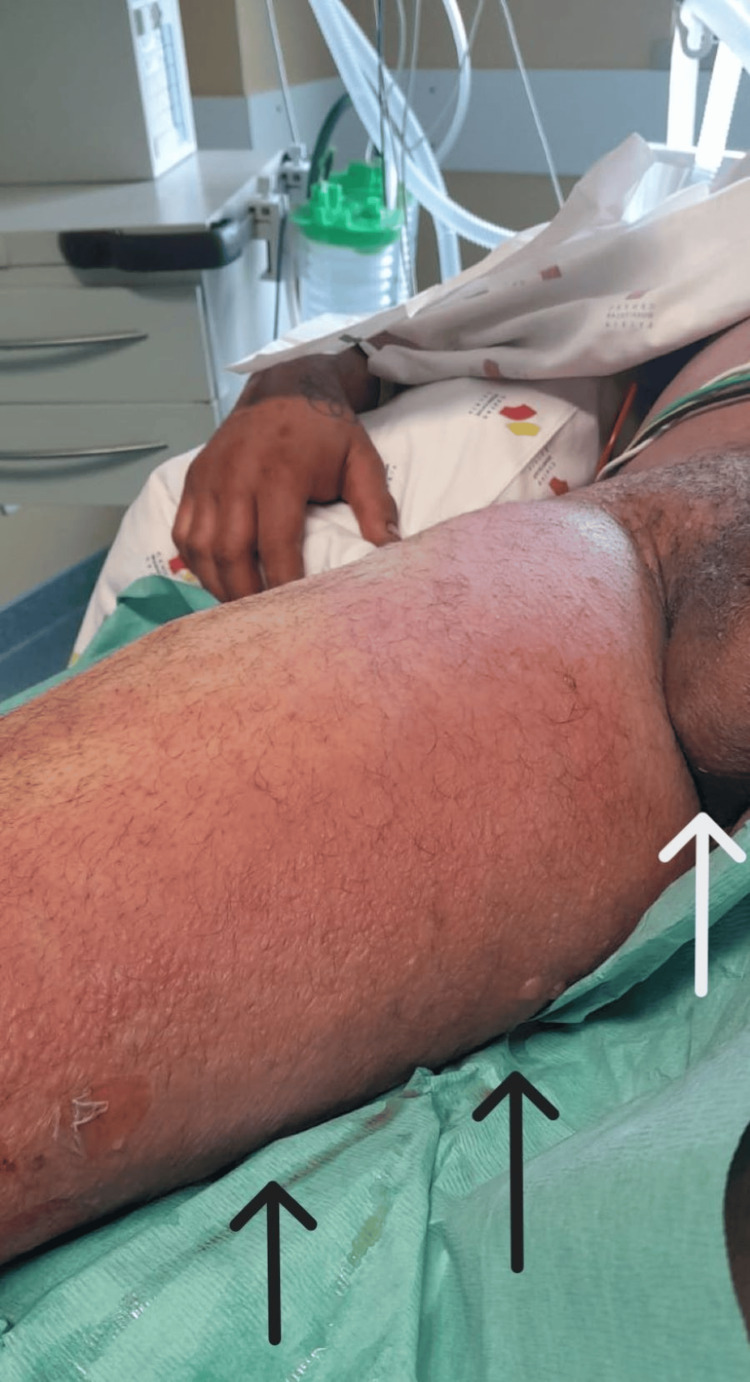
Progression in inflammatory signs to the medial region of the right thigh (black arrows) with extension to the scrotum (white arrow).

**Figure 8:**
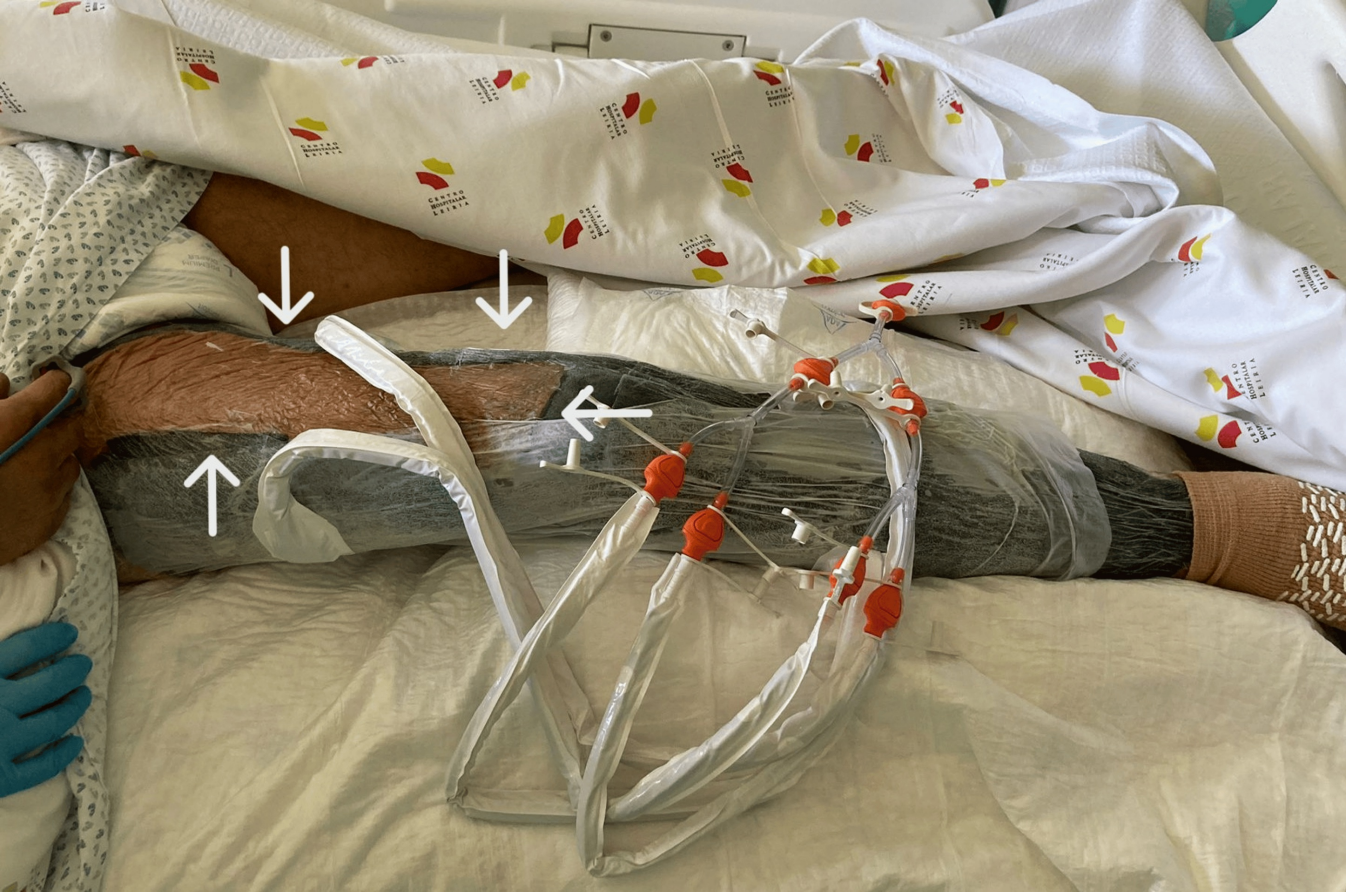
Right leg showing significant improvement in inflammatory signs (white arrows), still under negative pressure treatment.

Over the following two days, the patient required additional surgeries due to the progression of the necrotizing fasciitis to the upper lateral region of the right thigh (Figure 6). By the fourth day, inflammatory signs were also present in the medial region of the right thigh with extension to the scrotum (Figure 7). It was decided to transfer the patient to a tertiary hospital with hyperbaric oxygen therapy (HBOT) capability. The patient was transferred while ventilated, under renal replacement therapy, and still on norepinephrine at 0.8 μg/kg/min and vasopressin on the fifth day of hospitalization. He remained at the tertiary hospital for six days, during which he underwent five HBOT sessions and one additional surgery. He was then transferred back to our hospital, with significant improvement in inflammatory signs in his right lower limb (Figure 8).

Two days after the transfer, he was extubated and no longer required vasopressor support. Two days later, renal replacement therapy was discontinued. Clindamycin was discontinued after 10 days of treatment, and piperacillin/tazobactam was de-escalated to ceftriaxone after 11 days, which he continued for eight more days. The patient was transferred to the Plastic Surgery ward with the diagnosis of invasive GAS infection with TSS and necrotizing fasciitis and subsequently underwent skin grafting. He was discharged one month later to a rehabilitation center. The patient continues to be followed up by Plastic Surgery and Rehabilitation Medicine. Approximately one year after his hospitalization, the patient is autonomous and has returned to an active adapted lifestyle, including part-time work. He developed a scar formation process with complications, requiring a new graft, which is still in the healing phase.

## Discussion

Sepsis is a serious clinical syndrome that is associated with high morbidity and mortality rates for patients suffering from it. It is nowadays considered a major public health problem, as it is estimated that it costs more than $20 billion annually for US healthcare systems [5]. Various biomarkers for inflammation have been proposed, yet none have proven sufficient for the early, specific, and accurate diagnosis of systemic inflammation [6].

The incidence of GAS infection has been increasing, but in most cases, it is associated with mild disease. Invasive disease is rare, but it carries high morbidity and mortality, making a high level of suspicion crucial for an appropriate approach [2,3,7-9].

There are numerous risk factors for invasive disease, including HIV infection, intravenous drug use, diabetes mellitus or other forms of immunosuppression, malignancies, burns, trauma, postpartum status, obesity, other viral infections, homelessness, recent surgery, cardiac disease, peripheral vascular disease, and the use of corticosteroids or NSAIDs [10-13]. TSS is caused by the expression of bacterial toxins, which trigger an overwhelming systemic inflammatory response, characterized by fever, shock, and multi-organ failure [2,14]. The diagnostic criteria for TSS include hypotension and multi-organ involvement, confirmed by the isolation of GAS from a normally sterile site [15]. Treatment of TSS requires early recognition, as does the management of necrotizing soft tissue infections, due to the high risk of mortality [13,16,17].

The term "fasciitis" often leads to the misconception that the fascia or muscle aponeurosis must be involved, but in fact, the most common site of infection is the superficial fascia [18]. The diagnosis can be challenging and is often mistaken for cellulitis in its early stages, with delayed diagnosis potentially leading to a significant increase in mortality [18,19]. Certain clinical signs can aid in the early recognition of this condition, such as the appearance of cutaneous necrosis or bullae, ecchymosis, subcutaneous emphysema, and altered mental status, among others [18]. Several diagnostic scores for necrotizing fasciitis have been proposed, but none are universally accepted due to their low sensitivity [19]. Surgical intervention is crucial for improving patient outcomes and should be performed early (within 12 hours). Therefore, early multidisciplinary intervention can be a key factor in patient survival [18,19].

In addition to surgical treatment, antibiotic therapy is essential. Empirical treatment should include broad-spectrum coverage, encompassing gram-negative bacteria based on local resistance patterns, as well as methicillin-resistant *Staphylococcus aureus* (MRSA) coverage. In cases of renal dysfunction, linezolid or daptomycin may be effective alternatives to vancomycin [19]. Clindamycin or linezolid should also be included due to their ability to inhibit toxin production, which is responsible for much of the pathophysiology of invasive GAS disease, particularly in the presence of TSS [2,19,20]. There are no studies clearly defining the ideal duration of antibiotic therapy; however, it is generally recommended to continue antibiotics until surgical debridement is no longer necessary and the patient shows significant improvement and has been afebrile for at least 48 hours [19].

The administration of intravenous immunoglobulin is also an appropriate therapy in the presence of TSS, as it appears to significantly reduce its high mortality rate [19,21,22]. This treatment seems to enhance bactericidal activity by facilitating bacterial opsonization, neutralizing superantigens and toxins, stimulating leukocytes, and promoting a generalized anti-inflammatory effect [2].

HBOT is a medical treatment that delivers 100% oxygen at a pressure of 2-3 absolute atmospheres [2,19]. In necrotizing fasciitis, the tissues are hypoxemic, reducing the production of free radicals and the phagocytic capacity of neutrophils [2]. HBOT significantly increases dissolved oxygen in the blood, leading to better oxygenation of the affected tissues. In addition to improving leukocyte function, it also enhances antibiotic activity, reduces toxin production, and inhibits the growth of anaerobes [19]. Although theoretically an excellent strategy, the role of this treatment in necrotizing fasciitis has been debated due to the lack of randomized studies demonstrating its benefit. Nonetheless, it appears to be beneficial and, without delaying other treatments, should be considered an adjunctive therapy [2,19].

## Conclusions

Invasive GAS disease, although rare, has very high morbidity and mortality rates. Its recognition requires a high level of awareness of the condition and is essential for the early involvement of the necessary specialties. The patient's management should include broad-spectrum and anti-toxin antibiotic therapy, along with surgical debridement. Intravenous immunoglobulin has also shown benefits, and while HBOT appears to be advantageous, it should not delay other treatments, particularly surgical debridement.

This article describes the clinical case of a young man with no identified risk factors or entry points who developed an invasive GAS infection, rapidly progressing to necrotizing fasciitis and TSS. The swift intervention, including invasive organ support, antibiotic therapy, repeated surgical debridement, and intravenous immunoglobulin, was likely decisive in the patient's favorable outcome. Nevertheless, there was a significant improvement with HBOT, although it is difficult to assess its isolated benefit. It is also important to emphasize that despite all the early interventions, the patient experienced high morbidity due to the need for multiple surgical debridements. One year after the event, the patient is still undergoing skin graft treatments and has not yet fully returned to normal life. On the other hand, his renal function has returned to normal, and he does not appear to have any other significant sequelae.
